# Anti-Allergic Cromones Inhibit Histamine and Eicosanoid Release from Activated Human and Murine Mast Cells by Releasing Annexin A1

**DOI:** 10.1371/journal.pone.0058963

**Published:** 2013-03-18

**Authors:** Samia Yazid, Ajantha Sinniah, Egle Solito, Virginia Calder, Rod J. Flower

**Affiliations:** 1 William Harvey Research Institute, Barts and The London School of Medicine, Queen Mary University of London, London, United Kingdom; 2 Division of Molecular Therapy, Institute of Ophthalmology, London, United Kingdom; French National Centre for Scientific Research, France

## Abstract

**Background and Purpose:**

Although the ‘cromones’ (di-sodium cromoglycate and sodium nedocromil) are used to treat allergy and asthma, their ‘mast cell stabilising’ mechanism of pharmacological action has never been convincingly explained. Here, we investigate the hypothesis that these drugs act by stimulating the release of the anti-inflammatory protein Annexin-A1 (Anx-A1) from mast cells.

**Experimental approach:**

We used biochemical and immuno-neutralisation techniques to investigate the mechanism by which cromones suppress histamine and eicosanoid release from cord-derived human mast cells (CDMCs) or murine bone marrow-derived mast cells (BMDMCs) from wild type and Anx-A1 null mice.

**Key results:**

CDMCs activated by IgE-FcRε1 crosslinking, released histamine and prostaglandin (PG) D_2_, which were inhibited (30–65%) by 5 min pre-treatment with cromoglycate (10 nM) or nedocromil (10 nM), as well as dexamethasone (2 nM) and human recombinant Anx-A1 (1–10 nM). In CDMCs cromones potentiated (2–5 fold) protein kinase C (PKC) phosphorylation and Anx-A1 phosphorylation and secretion (3–5 fold). Incubation of CDMCs with a neutralising anti-Anx-A1 monoclonal antibody reversed the cromone inhibitory effect.

Nedocromil (10 nM) also inhibited (40–60%) the release of mediators from murine bone marrow derived-mast cells from wild type mice activated by compound 48/80 and IgE-FcRε1 cross-linking, but were inactive in such cells when these were prepared from Anx-A1 null mice or when the neutralising anti-Anx-A1 antibody was present.

**Conclusions and Implications:**

We conclude that stimulation of phosphorylation and secretion of Anx-A1 is an important component of inhibitory cromone actions on mast cells, which could explain their acute pharmacological actions in allergy. These findings also highlight a new pathway for reducing mediator release from these cells.

## Introduction

Disodium cromoglycate was the first cromone anti-allergic agent to be discovered but since its introduction into clinical medicine some 50 years ago [1], other cromones or ‘cromoglycate-like’ drugs have been developed including nedocromil, lodoxamide, traxanol and amlexanox. Some H_1_ antagonists (e.g. ketotifen, azelastine, pemirolast and olopatidine) also appear to share a similar pharmacology (or exhibit cross-tachyphylaxis) with cromoglycate [2]. Most of these drugs are used for the routine treatment of mild to moderate asthma and/or the topical treatment of ocular and other allergic symptoms. Cromoglycate is also used for treating intestinal inflammation [3], [4].

The cromoglycate-like drugs can inhibit both the early and the late phase of the asthmatic reaction in man [5], [6] as well as allergic ‘asthma’ or pulmonary inflammation in animal models of the disease [7]–[13]. Their anti-asthmatic activity is attributed to their anti-inflammatory properties by most authorities [14]–[16].

Although the prototype drug, cromoglycate, was developed in the 1960s the exact mechanism of action of this group has proved elusive. Early experiments [1], [17]–[20] led to the concept that these drugs acted mainly on mast cells to suppress histamine release, but they also inhibit cytokine generation [21]. The cromones are also effective in other models of inflammation [22]–[26] and influence many facets of the inflammatory process *in vivo* [27] or *in vitro*, e.g. eicosanoid generation [28], [29], which are unrelated to mast cell activation. Any putative mechanism of action must embrace this diverse pharmacology.

Anx-A1 is a 37kDa member of the annexin super-family (13 proteins in mammals). Both the full length protein and its N-terminal peptide *N*-acetyl*_2-26_*, have been shown by us, and by other laboratories to possess powerful anti-inflammatory actions in a wide variety of animal models of acute and chronic inflammation [30] including inhibition of histamine release in allergic pleuritis [31] and cytokine release in murine models of asthma [32]. Absence or degradation of the protein has also been implicated in the pathogenesis of asthma and airway hyperactivity [33], [34].

Anx-A1 is present in many differentiated cell types in man and animals but is particularly abundant in cells of the myeloid lineage including neutrophils, eosinophils, macrophages and mast cells. Glucocorticoids (GCs) potently stimulate synthesis and secretion [35]–[38] of Anx-A1 and there is a correlation between plasma corticosterone/cortisol and peripheral blood Anx-A1 concentrations [38], [39]. Not only do GCs increase the transcription of the Anx-A1 gene in target cells including mast cells [40] but also stimulate the release of pre-existing cytosolic pools of Anx-A1 through a receptor-dependent, non-genomic mechanism. This GC-induced secretory event is preceded by phosphorylation at Ser^27^ (and other sites), apparently as a result of PKC activation [41]–[43] by a glucocorticoid receptor (GR)-dependent mechanism. This is crucial for Anx-A1 release as the *Ser^27^*-*Ala^27^* mutant cannot be secreted by cells and has a different intracellular distribution [44]. Once on the cell surface, Anx-A1 can act in an autocrine (or paracrine) fashion to inhibit cell activation by interaction with receptors of the formyl peptide receptor (FPR) family, specifically FPR-L1, also now known as FPR2 or ALXR in man [45]–[48].

We have recently reported that the ability of cromones to inhibit PMN leukocyte activation [49] and eicosanoid release by U937 cells [50] depends upon their ability to release Anx-A1. This is secondary to a potentiation of PKC activity caused by an inhibitory action by cromones on the intracellular protein phosphatase 2A (PP2A), which normally limits the action of PKC.

Here, we report that a similar mechanism also accounts for the acute inhibitory effect of these drugs on histamine and eicosanoid secretion by human and murine mast cells. This not only provides a mechanistic explanation for the acute pharmacological action of these 50-year-old drugs but also gives a clue to a new pathway whereby the release of mediators from mast cells can be modulated.

## Methods

### Cord-derived human mast cell culture

We used the protocol of Dahl *et al* [51]. Briefly, commercially available CD34^+^ stem cells were cultured for 2 weeks in StemSpan (StemCell Technologies, Grenoble, France) serum-free medium supplemented with 100 ng/ml human SCF, 50 ng/ml IL-6 and 1 ng/ml IL-3 (added during the first 14 days) and 100∶g/ml penicillin/streptomycin (Peprotech, London, UK). The medium was replenished with 10% FCS from week 8.

After 10 weeks, cells were assessed for cytoplasmic granule appearance using toluidine blue staining, and expression of both FcεRI and c-Kit was assessed by flow cytometry. Culture was continued until greater than 95% of evaluated cells were identified as CDMCs and the required cell numbers for experiments were reached. Cells were used for experiments between 11 and 18 weeks of culture.

### Murine bone marrow derived mast cells

To generate BMDMCs, femur bones from Anx-A1^+/+^ or Anx-A1^-/-^ BALB/c mice (4-6 weeks old, Charles River, Kent, UK) were isolated and progenitor cells were flushed out using a sterile protocol and cultured in RPMI1640 (Invitrogen, Paisley, UK) containing 10%FBS, 4 mM glutamine, 100 units/ml of penicillin, 100 µg/ml of streptomycin, 0.1 mM non-essential amino acids, and 50 µM 2-mercaptoethanol together with 5 ng/ml of r-murine IL-3 and 10 ng/ml SCF (PeproTech, London, UK).

After 4–5 weeks of culture, cells were assessed for cytoplasmic granule appearance using toluidine blue staining, and expression of both FcεRI and c-Kit was assessed by flow cytometry. Culture was continued until greater than 95% of evaluated cells were identified as BMDMCs and the required cell numbers for experiments were reached.

Animal work was performed according to UK Home Office regulations (Guidance on the Operation of Animals, Scientific Procedures Act, 1986) and was approved by the Queen Mary University of London Ethics Committee (London, UK). Human cells were prepared according to a protocol approved by the East London & the City Local Research Ethics Committee (no. 06/Q605/40; P/00/029 ELCHA, London, UK).

### IgE/anti-IgE activation of CDMCs/BMDMCs

Aliquots of CBMCs were activated by crosslinking surface IgE with an anti-IgE antibody. Cells were incubated overnight with azide-free IgE (4 µg/mL) to pre-sensitise the cells and the following day they were activated by adding anti-IgE antibody (25 µg/mL; Serotec, Oxford, UK). Cell-free supernatants were taken at 1 h to measure histamine and/or PGD_2_ release. Aliquots were stored at –70°C for subsequent analysis.

In the case of BMDMCs, cells were incubated overnight with anti-mouse monoclonal dinitrophenyl (DNP) - IgE (100 ng/mL; Sigma) to sensitise the cells and the following day they were activated by adding DNP-BSA (1 µg/mL; Sigma-Aldrich, Dorset, UK). Cell-free supernatants were collected at 1h to measure histamine and/or PGD_2_ release. Aliquots were stored at –70°C for subsequent analysis.

When drugs or antibodies were tested, these were added to cells 5 min prior to IgE cross-linking.

### 48/80 challenge of BMDMCs

Aliquots of BMDMCs were stimulated with Compound 48/80 (10 µg/ml; Sigma-Aldrich, Dorset, UK) for 10 min at 37°C. Drugs or antibodies were added to the cells 5 min prior to challenge. Cell-free supernatants were taken to measure histamine and/or PGD_2_ release. Aliquots were stored at –70°C for subsequent analysis.

### Drugs used

In previous experiments [50] we noted that glucocorticoids (GCs) stimulated PKC and Anx-A1 phosphorylation through a GR-dependant mechanism, and that the known PP2A inhibitor, okadaic acid, potentiated this effect. For this reason, we included these drugs alongside the anti-allergics cromoglycate, nedocromil in our experiments. In one experiment, we also tested ketotifen, a drug unrelated chemically to the cromone structure, but which shares some cromone pharmacology [2].

In some cases the drug-treated cell aliquots were incubated with a well characterised (see; [30]) specific neutralising anti-Anx-A1 (clone 1B; 20 µg/mL), or isotype matched irrelevant (IgG1, ABD Serotec, Oxford, UK), mabs.

### Measurement of histamine release

A commercially-available enzyme immunoassay was used to detect and quantify histamine-released in the supernatant (SPI bio, Strasbourg, France). The assay was conducted following the manufacturer’s protocols. A standard curve ranging from 0.39–50 nM histamine was prepared using the reagent provided and the optical density was then read within 60 min in a microplate reader (Titertek™, Vienna, Austria) at 405 nm. In some cases, the total cell content of histamine was established by freeze-thawing of cells prior to challenge.

### Measurement of PGD_2_ release

A commercially-available enzyme immunoassay (Cayman Chemical, Michigan, USA) was used to detect and quantify PGD_2_ released in the supernatant. The assay was conducted following the manufacturer’s protocols. A standard curve ranging from 78–10,000 pg/ml PGD_2_ was prepared using the reagent provided and the optical density was then read within 60 min in a microplate reader (Titertek™, Vienna, Austria) at 405 nm.

### ELISA for Anx-A1

Anx-A1 protein levels in conditioned medium were determined by ELISA as reported by Goulding *et al* [36]. Briefly, 96-well flat-bottomed ELISA plates (Greiner, Gloucestershire, UK) were coated with 1 µg anti-Anx-A1 mAb 1B in bicarbonate buffer (pH 9.6) and incubated overnight at 4°C. After washing in the bicarbonate buffer, potentially uncoated sites were blocked with 100 µL of PBS containing 1% BSA for 1h at room temperature. Sample aliquots (100 µL) or Anx-A1 standard solutions (prepared in 0.1% Tween-20 in PBS; concentration ranging between 10 and 0.001 µg/mL) were added for 1h at 37°C. After extensive washing in PBS/Tween-20, 100 µL of a polyclonal rabbit anti-human Anx-A1 serum (Zymed, Invitrogen, Paisley, UK; diluted 1∶1000 in PBS/Tween-20) was added (1h at 37°C) prior to incubation with donkey anti-rabbit 1gG conjugated to alkaline phosphatase (1∶1000; Sigma). The colour was developed by addition of 100 µL p-nitrophenyl phosphate (1 mg/mL in bicarbonate buffer, pH 9.6). Absorbance was read at 405nm (with a 620-nm reference filter) in a microplate reader (Titertek™, Vienna, Austria). Anx-A1 levels in the study samples were read against the standard curve and expressed as ng/ml.

### Collection of cellular material for analysis

For assessment of drug effects on protein phosphorylation, test compounds were pre-incubated for 5 min with aliquots of 2×10^5^ CDMCs cultured in 10% FCS medium. To analyse proteins of interest, CDMCs in suspension following drug treatment, were decanted into 1.5 ml Eppendorf tubes and gently centrifuged (2000 RPM) for 5 min. The supernatant was removed and reserved and the resultant pellet resuspended in 500 µl of lysis buffer containing 1 mM EDTA (which removes Anx-A1 attached to the cell membranes), 200 mM NaCl, 20 mM Tris-HCl (pH 8.0), 1 mM protease and 1 mM phosphatase inhibitors (equimolar mixture of Na_3_VO_4_, β-glycerophosphate, NaF). Both the cell supernatant and the cell lysate were analysed.

### Assessment of Ser^27^-Anx-A1-P and PKC activation

CDMC extracts were prepared as described above. The total cellular protein was determined and extracts analysed by conventional western blotting techniques. Immunodetection was accomplished using different antibodies recognizing either the full-length Anx-A1 protein, (polyclonal anti-Anx-A1 antibody; 1∶1000, Invitrogen Ltd, Paisley, UK) or Anx-A1 phosphorylated on *Ser^27^* (polyclonal anti-*Ser^27^*-Anx-A1 antibody; 1∶1000, Neosystems, Strasbourg, France).

PKC phosphorylation was assessed using polyclonal anti-phospho-PKC antibody (1∶1000; Cell Signaling Technology; New England Biolabs UK Ltd, Hitchin, UK). Total PKC was estimated using a polyclonal pan-specific anti-PKC antibody (1∶1000; Cell Signaling Technology; New England Biolabs UK Ltd, Hitchin, UK) and α-tubulin using a monoclonal anti-α-tubulin (1∶5000; Sigma-Aldrich, Poole, UK). Activated PKC isoforms were assessed using a panel of specific anti-phospho PKC antisera including PKC δ (Thr^505^), PKC ? Thr^538^, and PKC α/β Thr ^638/641^ antisera (1∶1000; Cell Signaling Technology; New England Biolabs UK Ltd, Hitchin, UK).

A horseradish peroxidase-conjugated secondary antibody (1∶2000; Sigma-Aldrich, Poole, UK) detected bands related to the proteins of interest and these were revealed by enhanced chemiluminescence.

### Drugs and reagents

Nedocromil sodium was generously supplied by Sanofi-Aventis (Paris, France) and cromoglycate sodium, dexamethasone phosphate, okadaic acid and ketotifen were obtained from Sigma-Aldrich (Poole, Dorset, UK). The drugs were made freshly on the day for every experiment. Human recombinant protein Anx-A1 was a gift from our colleague Dr F. D’Acquisto.

### Statistical analysis

Unless otherwise stated, *In vitro* analyses were repeated at least 3 times with distinct mast cell preparations. Values are expressed as mean ± SEM of *n* observations. Statistical differences between the treated groups were assessed by analysis of variance (ANOVA) followed by Bonferroni’s test for intergroup comparisons. A threshold *P* value ≤ 0.05 was taken as significant.

## Results

### Effect of cromones, dexamethasone and human recombinant Anx-A1 on histamine and PGD_2_ release from CDMCs

We first established that our cultured and sensitised human CDMCs responded with a release of histamine and PGD_2_ when challenged with IgE/anti-IgE and that this could be inhibited by the standard cromone, sodium cromoglycate.

**Figure 1:**
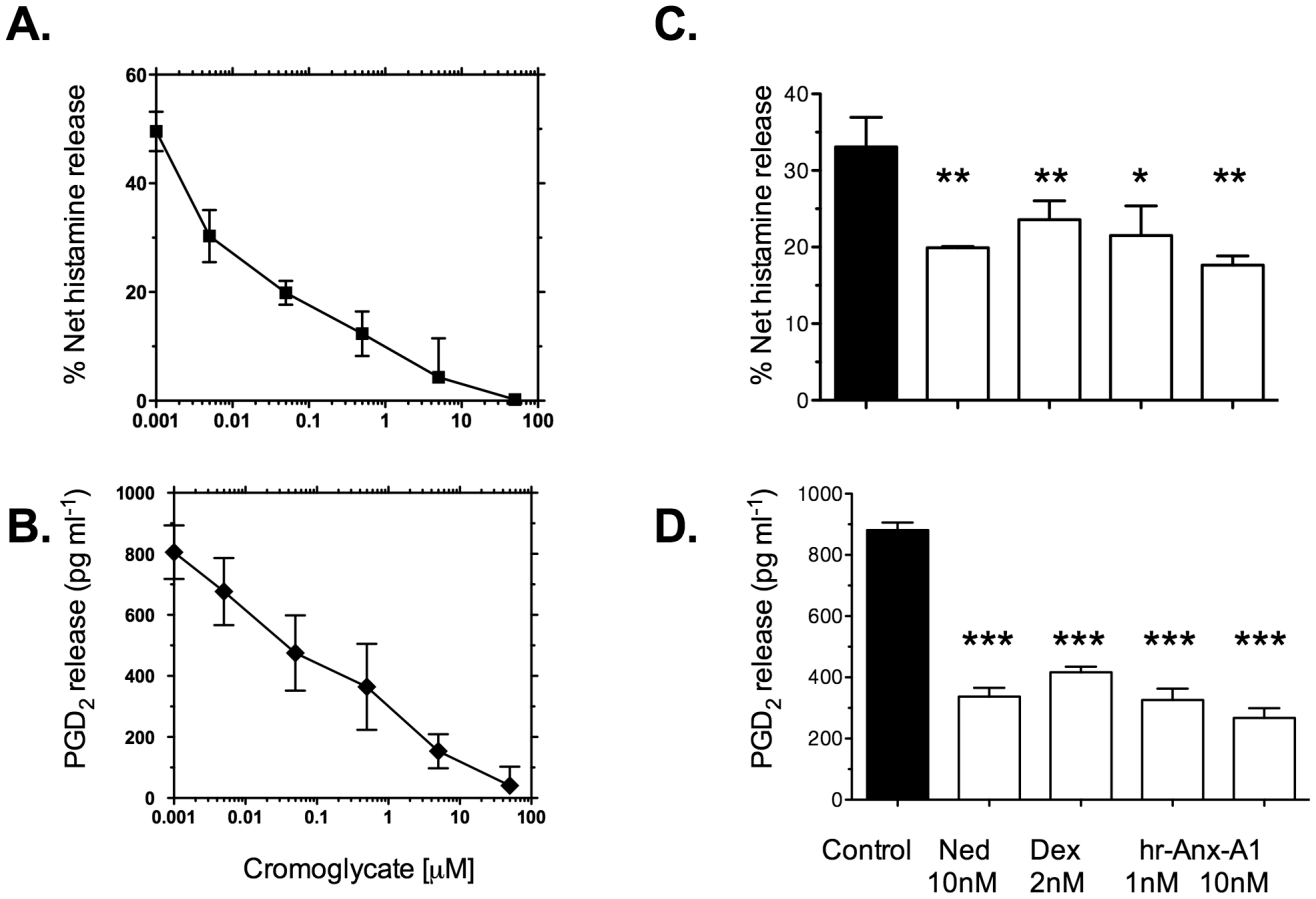
Cromoglycate, nedocromil, dexamethasone, and human recombinant Anx-A1 inhibit IgE/anti-IgE - induced histamine and PGD_2_ release from CDMCs. CDMCs were cultured, sensitised with IgE and challenged with anti-IgE for 1h as described. The cell culture supernatant was sampled and PGD_2_ release (pg/ml) or net % release of histamine was assessed by ELISA. Panels A and B. Cromoglycate 0.001–100 µM produces a concentration-dependent inhibition of histamine (panel A) or PGD_2_ (panel B) release. Each data point is expressed as the mean ± SEM (n = 3). Panels C and D. Vehicle (control), nedocromil (Ned; 10nM), dexamethasone (Dex; 2nM) or human recombinant Anx-A1 protein (hr-Anx-A1; 1 nM and 10 nM) was added to the wells to test their inhibitory effect on histamine (panel C) and PGD_2_ release (panel D). Each data point is expressed as the mean ± SEM (n = 3).* Signifies *P*<0.05; **P<0.01 and ***P<0.001 relative to vehicle treated cells.

Figure 1 (panels A and B) shows that 1h IgE cross-linking in CDMCs provoked a release of approximately 50% of intracellular histamine and 800 pg ml^-1^ PGD_2_. Cromoglycate produced a concentration-dependent inhibition of both histamine and PGD_2_ release with IC_50_ values of approximately 50 nM and 100 nM respectively.

We next compared the inhibitory action of the closely-related cromone, sodium nedocromil and the anti-inflammatory glucocorticoid dexamethasone (known to exert many effects through the release of Anx-A1) on the release of histamine and PGD_2_ from IgE/anti-IgE-challenged CDMCs and, since we hypothesise that these drugs act through the release of endogenous Anx-A1, we tested the action of human recombinant protein itself.

Figure 1 (panels C and D) shows that both nedocromil (10 nM) and dexamethasone (2 nM) also blocked histamine (approximately 42% and 29% respectively) and PGD_2_ (62% and 54% respectively) release at 5 min. In addition, hr-Anx-A1 at 1 nM and 10 nM inhibited histamine (approximately 25%, 47% respectively) and PGD_2_ release (approximately 63%, 68% respectively) to a similar extent as these anti-allergic drugs.

As nedocromil had very similar effects to cromoglycate, whilst being more potent, we chose to use this drug as the main cromone in subsequent experiments.

### Phosphorylation and secretion of Anx-A1 by CDMCs

In our previous work with U937 cells [50] and polymorphonuclear cells [49], we observed that cromones activated PKC and thereby promoted Anx-A1 phosphorylation. We demonstrated that this was an indirect action, probably secondary to inhibition of PKC de-phosphorylation by PP2A. We therefore checked to see if incubation with cromones promoted activation of PKC and phosphorylation and secretion of Anx-A1 from CDMCs in line with the effects we previously reported.

**Figure 2:**
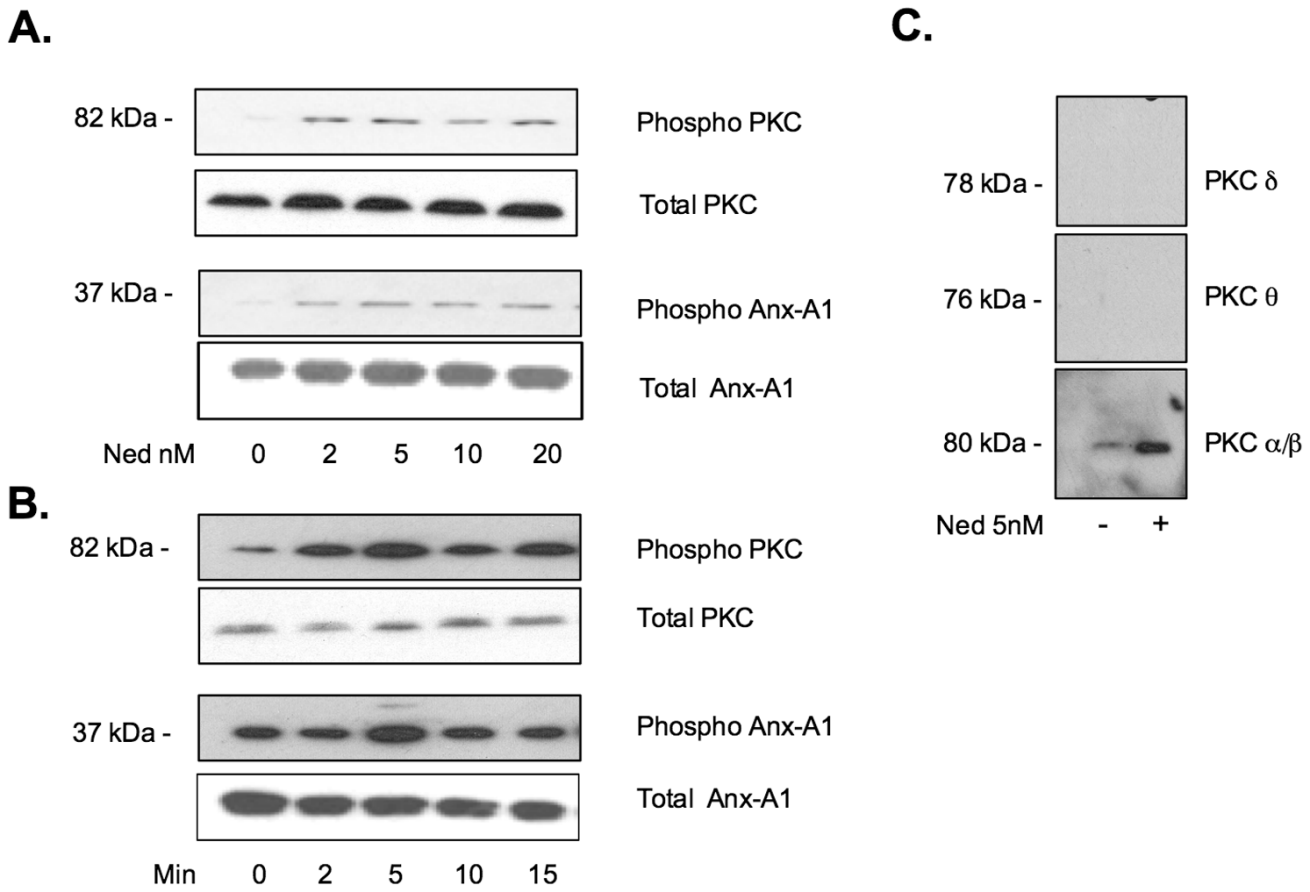
Nedocromil activates PKC_α/β_ and promotes Anx-A1 phosphorylation. Panel A. Aliquots of 2×10^5^ CDMC cells were cultured as described and incubated for 5 min with vehicle alone or escalating concentrations of nedocromil (Ned; 2-20 nM). The cell medium was then harvested and the cells lysed. Western blotting was used to assess the intracellular concentration of phospho-PKC and Ser^27^-phopsho-Anx-A1. A maximum effect was seen at 5 nM nedocromil at this time point. A representative blot from duplicate experiments. Panel B. Using a similar methodology, the time course of activation of PKC was assessed using nedocromil (Ned; 10 nM). A maximum effect was seen at 5 min treatment with the drug at this concentration. A representative blot from duplicate experiments. Panel C. Using samples of lysate prepared from CDMC cells treated with nedocromil (Ned; 5 nM) for 5 min, the relative abundance of three activated isoforms of PKC was assessed using specific antisera. Only PKC α/β was increased by nedocromil treatment. Activated PKC δ oρ θ isoforms were not detected in resting or stimulated cells under these experimental conditions.

Figure 2 panel A, shows the effect of escalating concentrations (0–20 nM) of nedocromil on the amount and disposition of phospho-PKC and Ser^27^-phospho Anx-A1 in CDMCs cultured in StemSpan serum-free medium.

In the untreated lane, small amounts of phospho-PKC and phospho Anx-A1 are detectable in the cytoplasm as well as in the media; this is a common finding as these cells are already partly ‘activated’ by the presence of stem cell factor in the medium.

Treatment with nedocromil produces an increase in the amount of phospho-PKC and phopsho-Anx-A1 in the cell with no further effect seen beyond 20 nM nedocromil at this time point. In panel B, nedocromil was used at a concentration of 10 nM and incubated with the cells for various time points (0–20 min). No further increase in phospho-PKC or phospho Anx-A1 was seen at time periods longer than 5 min. These parameters are in line with other data that we have obtained using (e.g.) U937 cells [50].

The MW of the phosphorylated PKC enzyme detected by Western blotting using the pan-specific PKC antibody was in the range 76–82 kDa suggesting the relevant isoform was either PKC δ (78 kDa), PKC θ (76 kDa) or PKC α/β (80–82 kDa). When we probed our blots with a panel of isoform specific anti-phospho PKC antisera (panel C), no PKC δ (Ser^643^) or PKC θ Thr^538^ was detectable however, the specific anti- phospho-PKC α/β Thr^638/641^ antibody showed good reactivity with a band that increased following nedocromil treatment. We therefore concluded that the main kinase responsible for Anx-A1 phosphorylation in the CDMC system was almost certainly PKCα/β.

### Effect of cromones on Anx-A1 secretion

We next determined whether treatment with cromones provokes Anx-A1 release from CDMCs into the cell medium.

**Figure 3:**
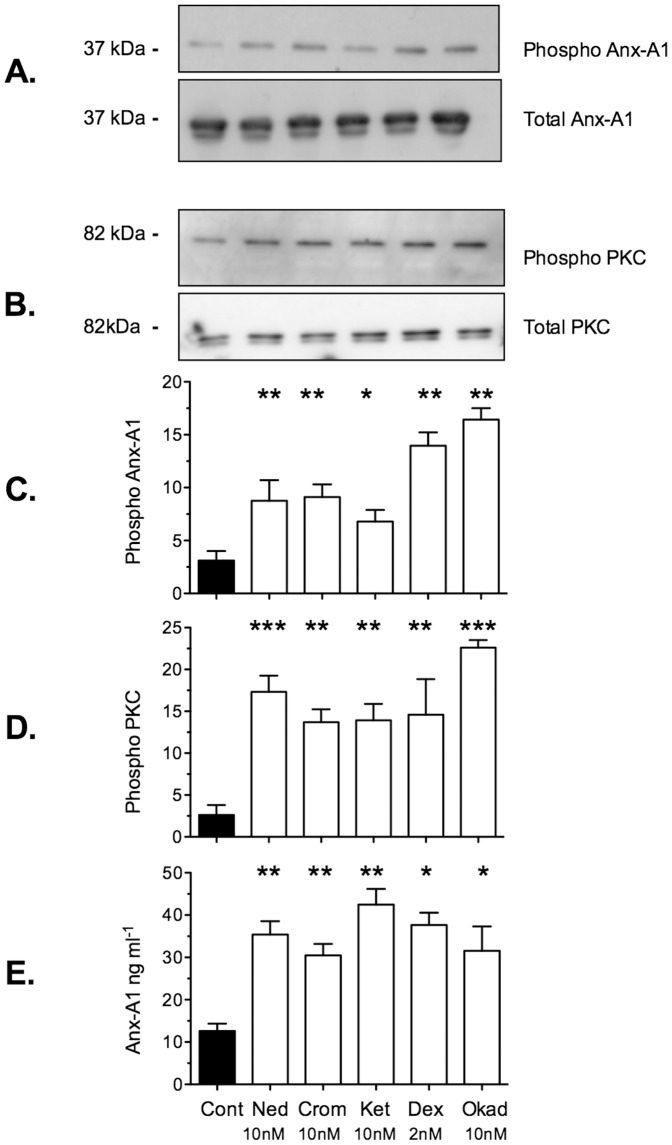
Cromones promote secretion of Anx-A1 from CDMC cells. Panels A and B. Aliquots of 2×10^5^ CDMC cells were cultured as described and incubated for 5 min with vehicle alone (‘control’), nedocromil (Ned; 10 nM), cromoglycate (Crom; 10 nM), ketotifen (Ket; 10 nM), dexamethasone (Dex; 2 nM) or okadaic acid (Okad; 10 nM). Anx-A1 (panel A) and PKC phosphorylation (panel B) were assessed by western blotting. There is no detectable change in the absolute intracellular amounts of these proteins at 5 min. Panels D and E. Densitometry data from 3 such experiments was analysed graphically in the corresponding bar graphs. Panel E. Anx-A1 released into the supernatant after incubation with the drugs was assessed using an ELISA assay and expressed as ng/ml culture fluid. * Signifies *P*<0.05; **P<0.01 and ***P<0.001 relative to vehicle treated cells.

Figure 3 panels A and B show that in addition to nedocromil (10 nM) itself, cromoglycate (10 nM), ketotifen (10 nM; another known ‘mast cell stabilising’ drug), dexamethasone (2 nM) and okadaic acid (10 nM; a known inhibitor of PP2A) potentiate the activity of PKC and promote additional Anx-A1 phosphorylation. Panels C and D show a densitometry analysis of 3 similar experiments and panel E shows that Anx-A1 is secreted into the cell culture fluid in response to these treatments, as assessed by ELISA assay.

These results show that all drug treatments tested significantly increased (∼2–5 fold) activated PKC and Ser^27^ phosphorylated Anx-A1 and increased the release of Anx-A1 into the cell culture fluid by ∼3–5 fold above that found under basal conditions.

### The acute inhibition of histamine and PGD_2_ release from CDMCs by nedocromil is Anx-A1 dependent

To ascertain the role of Anx-A1 in the mechanism of action of nedocromil, the effect of a specific neutralizing monoclonal anti-Anx-A1 antibody was tested.

**Figure 4:**
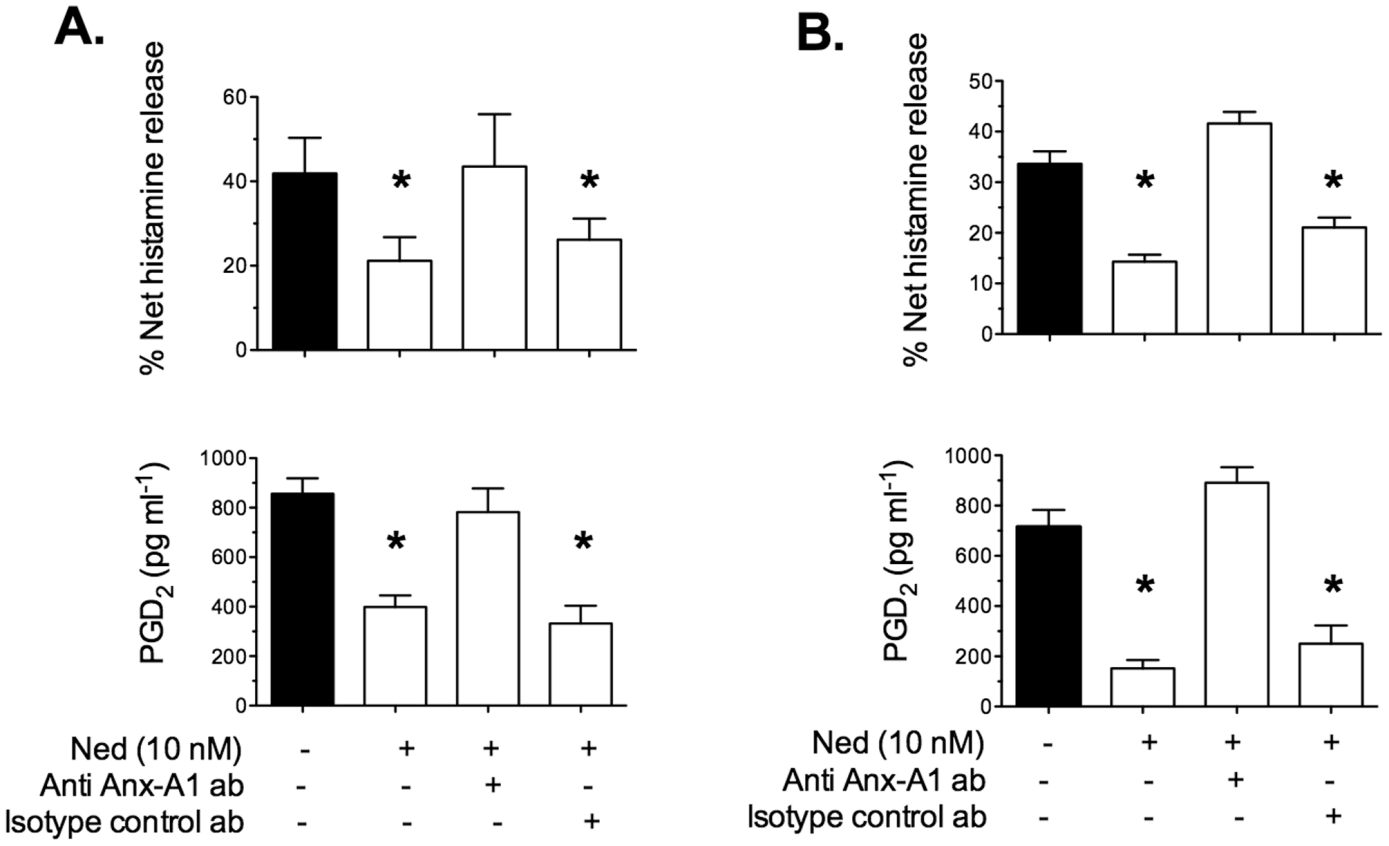
The inhibitory effect of nedocromil on IgE/anti-IgE-induced histamine and PGD_2_ release from is Anx-A1 dependent. Panel A. CDMCs were cultured, sensitised with IgE and challenged with an antigen as described. Nedocromil (Ned; 10 nM) was administered alone or in the presence of 10 µg/ml neutralising anti-Anx-A1 mab (or an irrelevant control) 5 min before challenge with anti-IgE. Histamine (upper bar chart) and PGD_2_ (lower bar chart) release into the cell culture fluid was assessed using ELISA as described above. Panel B. BMDMCs were prepared from wild type mice as described, and challenged with compound 48/80 as described. Nedocromil (Ned; 10 nM) was administered alone or in the presence of 10 µg/ml neutralising anti-Anx-A1 mab (or an irrelevant control) 5 min before challenge with anti-IgE. Histamine (top bar chart) and PGD_2_ (lower bar chart) release into the cell culture fluid was assessed using ELISA as above. The presence of neither the neutralising, nor the control monoclonal antibody had any effect on the release of mediators in the absence of nedocromil (data not shown). * Signifies *P*<0.05 relative to the appropriate control aliquots.

CDMCs were pre-incubated for 20 min with 20 μg/ml neutralizing Anx-A1, or irrelevant isotype-matched, monoclonal antibody and then incubated for 5 min with nedocromil sodium (10 nM). Figure 4 shows the effect of the co-incubation of CDMCs with neutralising anti-Anx-A1 (or irrelevant) mabs on the effect of inhibitory action of 10 nM nedocromil on histamine and PGD_2_ release. Under control conditions, the drug inhibited histamine and PGD_2_ release from IgE-activated CDMCs (panel A) as well as from 48/80-challenged CDMCs (panel B) by approximately 50% in both cases. This effect was completely abolished in the presence of the neutralising anti Anx-A1 mab (<6% inhibition). The irrelevant antibody was inactive. Neither the neutralising nor the irrelevant antibody alone had any significant effect on the release of mediators (data not shown).

### Effect of cromones on mediators released from BMDMCs from Anx-A1 wild type and null mice

The above experiments demonstrated that the acute effects of the cromone nedocromil in human CDMCs appeared to be Anx-A1 mediated. We next ascertained whether this was solely a feature of the cultured CDMC model that we used. To test this, we turned to the use of transgenic mice in which the Anx-A1 gene was globally deleted. Murine mast cells from wild type and Anx-A1 null mice were cultured and matured as described from bone marrow precursors.

We first used BMDMCs from wild type mice to establish whether these behaved in a similar fashion to the human CDMCs. Figure 4 panel B shows that nedocromil (10 nM) inhibits histamine (upper bar chart) and PGD_2_ (lower bar chart) release and that (as in the case of the CDMCs) this is blocked by immuno-neutralisation of secreted Anx-A1.

We next tested the inhibitory action of a range of 5 concentrations of nedocromil (0.5 – 10 nM) in two different protocols where mediator release was stimulated by DNP-IgE/DNP-BSA cross-linking and stimulation with compound 48/80.

**Figure 5:**
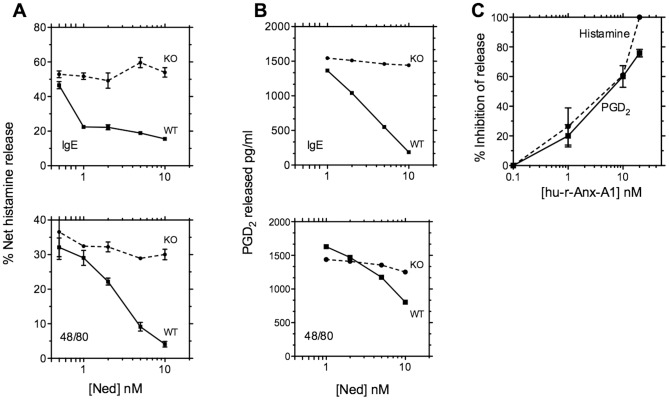
Nedocromil is inactive in BMDMCs from Anx-A1^-/-^ mice. Panel A. BMDMCs were prepared from Anx-A1^-/-^ and wild-type mice as described. Aliquots were sensitised with anti-DNP-IgE and challenged with DNP-BSA as described (upper graph) or stimulated with 10 µg/ml compound 48/80 (lower graph). Nedocromil was added in increasing concentrations (0.5 – 10 nM) and the release of histamine into the medium was assessed by ELISA. Results are expressed as % net histamine release; mean ± SEM; n = 2 with each sample assayed in triplicate. Panel B: BMDMCs were prepared from Anx-A1^-/-^ and wild-type mice as described. Aliquots were sensitised with anti-DNP-IgE and challenged with DNP-BSA as described (upper graph) or stimulated with 10 µg/ml compound 48/80 (lower graph). Nedocromil was added in increasing concentrations (1 – 10 nM) and the release of PGD2 into the medium was assessed by ELISA. Results are expressed as PGD2 released in pg/ml. Panel C. BMDMCs from Anx-A1^-/-^ mice retain their sensitivity to exogenous Anx-A1. BMDMCs were prepared from Anx-A1^-/-^ and aliquots were sensitised with anti-DNP-IgE and challenged with DNP-BSA as described, in the presence of escalating concentrations of human recombinant Anx-A1 (0.1–20 nM). Histamine and PGD_2_ release into the cell culture fluid was assessed by ELISA. The data is plotted as a concentration-inhibition curve. Each point was the mean of triplicates.

Figure 5 A and B show that nedocromil produces a concentration-dependent inhibition of histamine and PGD2 release, respectively, from wild type BMDMCs when this is elicited by either anti-DNP-IgE/DNP-BSA cross-linking in the case of pre-sensitised cells (panel A, upper bar chart), or compound 48/80 in un-sensitised cells (lower bar chart). However, the drug is without effect in the cells cultured from Anx-A1 null mice.

We also tested Anx-A1 in the cells lacking the Anx-A1 gene. Figure 5 (panel C) shows that whilst not being able to release Anx-A1, these cells retain their sensitivity to the protein which inhibits histamine and PGD_2_ release with an IC_50_ of approximately 5 nM.

## Discussion and Conclusions

Our data here clearly indicates that cromones, like the GCs, promote PKC activation and the subsequent phosphorylation, externalisation and release of Anx-A1 from CDMCs. The amounts of Anx-A1 released from CDMCs in this manner yield concentrations in the nM range, that are able substantially to reduce histamine and PGD_2_ release, as indicated by the experiments with r-hu-Anx-A1. Our data further suggests that this process is critical for the acute inhibitory action of the cromones in CDMCs and also murine mast cells derived from bone marrow precursors. That this mechanism is common to several cell types is suggested by our previous observations using U937 cells [50] and PMN [49], where a similar autocrine Anx-A1 dependent inhibition of cell function was observed following cromone treatment. We also report that dexamethasone, a glucocorticoid known to release Anx-A1 using this pathway (see; [30]), also inhibits histamine and PGD_2_ release in these cells.

Although not specifically investigated in this study, our previous work supports the notion that this effect is brought about following inhibition by these drugs of a phosphatase, probably PP2A, which secondarily prolongs the activation of PKC thereby further stimulating Anx-A1 phosphorylation and release.

It has always been clear that the cromones had a unique mechanism of pharmacological action. Their ‘mast cell stabilising’ effect [1], [17]–[20] is distinct from that of other drugs such as the β-agonists which, whilst being more efficient at ‘stabilising’ mast cells [52], do not share the characteristic spectrum of activity of the cromones.

There are a number of anomalous observations in the cromone literature that might be explained by the Anx-A1 dependent mechanism posited here. For example, there is a puzzling heterogeneity amongst species and between mast cell subtypes, in the actual ‘stabilising’ effect of these drugs and in the concentrations required to achieve this effect *in vitro* [53]–[55]. Indeed, a recent paper has questioned even whether cromoglycate, which is highly active in the rat, is active at all in the mouse [56]. This would imply a considerable species-variation in the response to this drug although we noticed that nedocromil worked well in our murine BMDMC preparation.

Since these drugs can only exert an appreciable effect on mediator release if Anx-A1 phosphorylation has already been triggered by another stimulus, such differences could reflect the relative degree of activation (and hence PKC stimulation/Anx-A1 phosphorylation) of cells from different sources or subjected to different experimental protocols. Perhaps this explains why the cromoglycate drugs have only a variable effect when given in the absence of an appropriate ‘priming’ stimulus that triggers this effect. This observation might also explain some of the anomalous dose-response relationships that have been reported [53], [57]. In this context it is interesting to note that the CDMCs cultured as described here are partly activated by the presence of SCF and that there is already a small pool of intracellular Ser^27^ phospho Anx-A1 present in these cells even before challenge with antigen or drug treatment.

The timing of cromone administration relative to the degranulating stimulus is also thought to be crucial to their effect [52] and these drugs exhibit strong tachyphylaxis [53], [58], [59]. Indeed, the refractory response that characteristically follows an application of these drugs, led to early speculation that the release of a labile rapidly-depleted anti-inflammatory substance [60] or other transient intracellular phenomenon [61], [62] may be involved in their action. Our results are consistent with such an interpretation and it is likely that the hypothesised ‘anti-inflammatory’ substance is actually Anx-A1.

Whilst our hypothesis, as presented here, is novel, there have been several previous observations that link cromone action to activation of signalling pathways and modification of potential down-stream molecular targets in mast cells. Treatment of mast cells with cromoglycate results in the phosphorylation of intracellular protein substrates including the erythrocyte band 4.1 group protein moesin [61], [63] and there have been scattered reports of an interaction between cromoglycate and PKC stretching back over some years e.g. [64]-[66]. Indeed Wang *et al* [67] previously investigated the possibility that these drugs inhibited PP2A, but were unable to detect an effect in their system. Other, earlier, workers had noted a correlation between the action of these drugs *in vivo* in a rat passive cutaneous anaphylaxis model and inhibition of another, alkaline, phosphatase [68], [69].

It is of interest that the anti-allergic drug ketotifen, a non-cromone member of this group appears to have similar actions on the Anx-A1 system. Ketotifen is a second-generation H_1_ antagonist that has (in common with some other members of this class) long been observed to have additional ‘mast cell stabilising properties’ [2]. It is interesting to speculate that all the H_1_ antagonists that have this additional action may have a secondary pharmacology as PP2A inhibitors and that this could be a useful screen to evaluate this property.

Recently there has been a resurgence of interest in the cromones and an alternative mechanism of action to that presented in this study, has been proposed by two groups [70], [71] who have suggested that the cromones may produce their therapeutic actions by acting through GPR35. This G-protein coupled receptor signals via the G*i* pathway. It is generally thought to be an orphan receptor although some have suggested that products of tryptophan metabolism such as kynurenic acid are the endogenous ligands, although relatively large concentrations of these substances are required to activate the receptor.

The first of these studies [71] used (CHO/HEK) cells transfected with this receptor and demonstrated that cromoglycate, nedocromil and zaprinast increased calcium mobilisation and inositol phosphate accumulation in these cells. There was a difference in specificity exhibited by the three drugs when tested on human, mouse and rat GPR35. In terms of potency, all the drugs were very similar.

Other authors [70] examined human and rat GPR35 in transfected HEK/yeast cells, using a β-arrestin-2 interaction assay. They identified a range of ligands including cromoglycate, zaprinast and dicoumarol. Cromolyn and zaprinast were full agonists; dicoumarol was a partial agonist.

GPR35 is apparently present in human mast cells (particularly when treated with IgE) as well as eosinophils and basophils although its relevance to asthma and allergy or to mast cell mediator release is not yet understood so it is unclear how these actions of the cromones could translate into therapeutic effects - or how they might integrate with the ‘Anx-A1 dependent’ mechanism we detailed here and elsewhere [49], [50]. However, it is worth noting that the effects that we report here occur within 5 min and that we have not looked at other actions of the cromones that may require a longer latency and which perhaps could be mediated by alternative GPR35 mechanisms.
